# Microorganisms and enzymes involved in polybutylene adipate terephthalate biodegradation

**DOI:** 10.1007/s00253-025-13565-4

**Published:** 2025-10-07

**Authors:** Miguel Fernandes, António A. Vicente, Andreia F. Salvador

**Affiliations:** 1 Centre of Biological Engineering, University of Minho, Campus de Gualtar, 4710-057 Braga, Portugal; 2 LABBELS–Associate Laboratory, Braga/Guimarães, Portugal

**Keywords:** Polybutylene adipate terephthalate, Biodegradation, Soil burial, Microorganisms

## Abstract

**Abstract:**

The European Union aims to achieve climate neutrality, protect the environment, and reduce marine litter, particularly caused by the pollution caused by recalcitrant plastics. The investment in biodegradable solutions as alternatives for conventional plastics is a priority for achieving a circular economy and tackling waste management challenges. However, the introduction of novel plastic blends in the environment must consider their effective biodegradation. Polybutylene adipate terephthalate (PBAT) is one of the most interesting polymers to integrate aliphatic–aromatic copolyesters as alternatives to recalcitrant plastics. Compared to other biodegradable plastics, PBAT offers superior flexibility and thermal stability. PBAT is degradable under industrial composting conditions, at thermophilic temperatures. At lower temperatures in natural environments, biodegradation is very challenging, but recent research has shown novel insights on PBAT biodegradation in a variety of environments. This review gives an overview of PBAT physical and chemical properties and its application in plastic blends and focuses on PBAT biodegradation. We compiled information about the microorganisms and enzymes involved in PBAT biodegradation and the environmental conditions in which the biodegradation occurs. For the first time, this review comprehensively compares enzymatic systems, microbial strains, and soil biodegradation while also identifying methodological limitations in the literature. The information presented herein is important to understand the opportunities and limitations of using PBAT in alternative plastic formulations and will hopefully guide the development of biotechnological solutions for plastic waste decontamination, contributing to building a greener future.

**Key points:**

• *The blend of PBAT with other materials, change their biodegradation potential*

• *Identification of the PBAT properties affecting biodegradation*

• *Compilation of the few microorganisms known as PBAT degraders in several environments*

## Introduction

Plastics have become crucial for modern society. Their specific characteristics, including durability, processability, and low production price, have led to their extensive use in varied applications worldwide since the middle of the twentieth century (Trinh Tan et al. 2008). Nonetheless, their persistence in the environment as trash on land and in drainage systems, eventually leaking into rivers and oceans, makes them a threat to natural ecosystems (Kedzierski et al. 2020; Pathak et al. 2014). An increasing amount is also ending up in landfills as part of municipal solid waste, with a considerable amount ending up contaminating the soil (Pathak et al. 2014). It is necessary to create worldwide waste management infrastructures to recover and recycle plastics. However, there are difficulties in plastic recovery and recycling due to several factors, such as plastic contamination with, for example, food residues (Arijeniwa et al. 2024; Flury and Narayan 2021). Consumers are becoming more aware of global plastic pollution, and with society awakening, the pressure and demand on the responsible entities as well as on companies to find sustainable alternatives and tackle the plastic crisis (Arijeniwa et al. 2024). The solution involves a multifaceted approach concerning prevention, reuse, recycling, recovery, and disposal. Within this policy, biodegradable plastics are key elements. Biodegradable plastics may offer more end-of-life routes (e.g., composting and anaerobic digestion) than the more resistant conventional plastics, while also being decomposed by microorganisms without harmful effects in the environment (Gómez and Michel, 2013; Kedzierski et al. 2020).

Biodegradable plastics include polyhydroxyalkanoates (PHAs), starches, and cellulose, and most formulations they are presented as plastic composites to attain the required properties. Polybutylene adipate terephthalate (PBAT) is a polymer widely used in plastic composites, for example, in food packaging and mulch films (Bilck et al. 2010; Muthuraj et al. 2014). PBAT represented 4.6% of the global bioplastic production capacity, with more than 113 million tonnes being produced in 2024 (https://www.european-bioplastics.org/market/#). The choice of PBAT lies on its physical and chemical properties, which confer good flexibility and elongation at break to the final product (Fu et al. 2020; Zhang and Sharaf 2024).

This review stands out by offering the most comprehensive and up-to-date synthesis of microbial and enzymatic mechanisms driving PBAT biodegradation, with a special emphasis on soil environments. Unlike previous studies, this work uniquely integrates comparative analyses of enzymatic systems, microbial strains, and environmental factors, while also critically addressing methodological gaps in the current literature. By bringing together fragmented findings, including the diversity of PBAT degraders, the influence of plastic blends/composites on biodegradability, and soil-specific microbial dynamics, this review delivers vital insights. Its integrative approach and identification of research limitations provide a strategic roadmap for future study that aims at advancing sustainable plastic solutions, improving biodegradable plastic performance, or investigating polymer biodegradation and waste management.

## Definition of biodegradation

The American Society for Testing and Materials (ASTM), the International Standardization Organization (ISO), and the European Committee for Standardization (CEN) have developed standardized definitions for the terms “biodegradable plastics” and “biodegradability” (Table 1). The definitions are all similar; however, only the CEN definition for biodegradable plastics indicates that the material needs to be converted to water, CO_2_, and/or methane and new cell biomass. The ISO and ASTM definitions only indicate a significant change in chemical structure under specific environmental conditions resulting in a loss of some properties that may vary as measured by standard test methods appropriate to the plastic and the application.

**Table 1:** Definitions used by different organizations (ASTM, CEN, and ISO) related to biodegradation

Organization		Definition
CEN	Biodegradable plastics	A degradable material in which the degradation results from the action of microorganisms and ultimately the material is converted to water, CO_2_ and/or methane and new cell biomass (Pagga 1998)
	Biodegradation	Degradation caused by biological activity, especially by enzymatic action, leading to a significant change in the chemical structure of a material (Pagga 1998)
	Inherent biodegradability	The potential of a material to be biodegraded, established under laboratory conditions (Pagga 1998)
	Ultimate biodegradability	The breakdown of an organic chemical compound by microorganisms in the presence of O_2_ to CO_2_, water and mineral salts of any other elements present (mineralization) and new biomass or in the absence of O_2_ to CO_2_, methane, mineral salts, and new biomass (Pagga 1998)
ASTM D883-18 (2018)—Standard Terminology Relating to plastics	Degradable plastic	A plastic designed to undergo a significant change in its chemical structure under specific environmental conditions resulting in a loss of some properties that may vary as measured by standard test methods appropriate to the plastic and the application in a period of time that determines its classification
	Biodegradable plastics	A degradable plastic in which the degradation results from the action of naturally occurring microorganisms such as bacteria, fungi, and algae
ISO 472 (2013) Plastics—Vocabulary	Degradable plastic	A plastic designed to undergo a significant change in its chemical structure under specific environmental conditions, resulting in the loss in some properties, as measured by standard test methods appropriate to the plastic and the application, in a given period of time that determines whether the plastic can be classified as biodegradable or not
	Biodegradable plastics	A plastic designed to undergo a significant change in its chemical structure under specific environmental conditions resulting in a loss of some properties that may vary as measured by standard test methods appropriate to the plastic and the application in a period that determines its classification. The change in the chemical structure results from the action of naturally occurring microorganisms
	Degradation	Irreversible process leading to a significant change in the structure of a material, typically characterized by a change of properties (e.g. integrity, molecular mass or structure, mechanical strength) and/or by fragmentation, affected by environmental conditions, proceeding over a period of time and comprising one or more steps
	Biodegradation	Degradation caused by biological activity, especially by enzymatic action, leading to a significant change in the chemical structure of a material
	Ultimate aerobic biodegradation	Breakdown of an organic compound by microorganisms in the presence of oxygen into carbon dioxide, water, and mineral salts of any other elements present (mineralization) plus new biomass

The term “bio-based” used commonly in the literature causes some misinterpretations. A bio-based plastic implies that the material is derived from biomass. Partially bio-based (or hybrid) plastics are synthesized with both renewable and conventional fossil fuel-based carbon (Atiwesh et al. 2021). It is important also to clarify the term bioplastic, which is often misused and misleading. Bioplastics are a group of polymers that are bio-based, biodegradable, or both (Atiwesh et al. 2021).

## PBAT synthesis, characteristics and applications

**Fig. 1:**
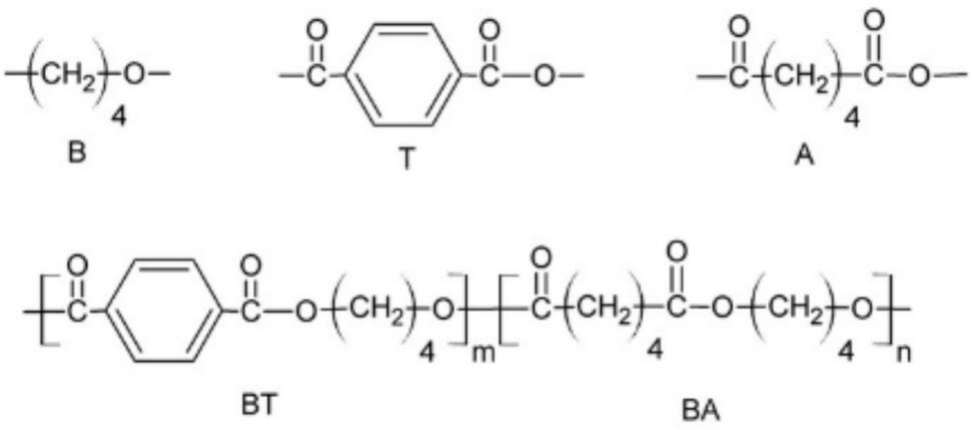
Structure of 1,4-butanediol (B), terephthalic acid (T), adipic acid (A), and PBAT (BT—butylene terephthalate; BA—butylene adipate) (Kijchavengkul et al. 2010a)

Polybutylene adipate terephthalate (PBAT) is a polymer synthesized through the polycondensation reaction of butanediol, adipic acid, and terephthalic acid, by using a common polyester production technology and equipment (Jian et al. 2020). The chemical structures of the different monomers are shown in Fig. 1. As polycondensation catalysts, tin, titanium, and zinc can be employed (Witt et al. 1995). The process of production consists of pre-mixing, pre-polymerization, and final polymerization. To eliminate water and to improve the condensation reaction, high temperatures and vacuum with extended reaction times are necessary (Witt et al. 1995). To improve the crystallization behaviour and prevent sticking, inorganic compounds are used as nucleating agents such as talc, chalk, mica, or silicon oxides in the final polymerization step. PBAT is not a bio-based plastic since it is partially produced by using fossil carbon sources, although some efforts are being made to produce all of the constituents from bio-based sources (Kruyer and Peralta-Yahya 2017; Tachibana et al. 2015; Yim et al. 2011).

Due to the aromatic unit in the molecule chain, PBAT presents some interesting mechanical properties, being more flexible than other polyesters, including polylactic acid (PLA) and polybutylene succinate (PBS), and has analogous mechanical properties to low-density polyethylene (LDPE) (Yamamoto et al. 2005), one of the most used and recalcitrant plastics. Some of the properties include a melting point of 115 to 125 °C, a crystallinity point of 60 °C, a tensile strength of 21 MPa, an elongation at break of 670%, a flexural strength of 7.5 MPa, and a melt flow index at 190 °C under 2.16 kg around 4, making it very appropriate for blowing film applications (Jian et al. 2020). Since PBAT has 2 different units and the mechanical properties are influenced by monomer composition and molecular weight, it can be tailored.

Packaging, particularly food packaging, is the main sector where PBAT is used (Moustafa et al. 2017). Several packages containing compostable PBAT-based materials are commercially available and are produced by companies such as BASF and Novamont. It is also used for agricultural purposes as mulch films, to increase crop yields because they can increase soil temperatures, maintain soil moisture, control weed development, and protect against adverse weather and pests (Souza et al. 2019). The disadvantage of PBAT is its high manufacturing cost and poor mechanical properties when compared to conventional nonbiodegradable polymers, which can reduce its commercial applications (Moustafa et al. 2017). This problem has been minimized by blending PBAT with other more cost-effective biodegradable polymers or adding fillers, compatibilizers, and plasticizers to create cost-effective composites to increase the applications and enhance the properties (Rocha et al. 2018; Wei et al. 2015).

## Biocatalysts in PBAT biodegradation

**Fig. 2:**
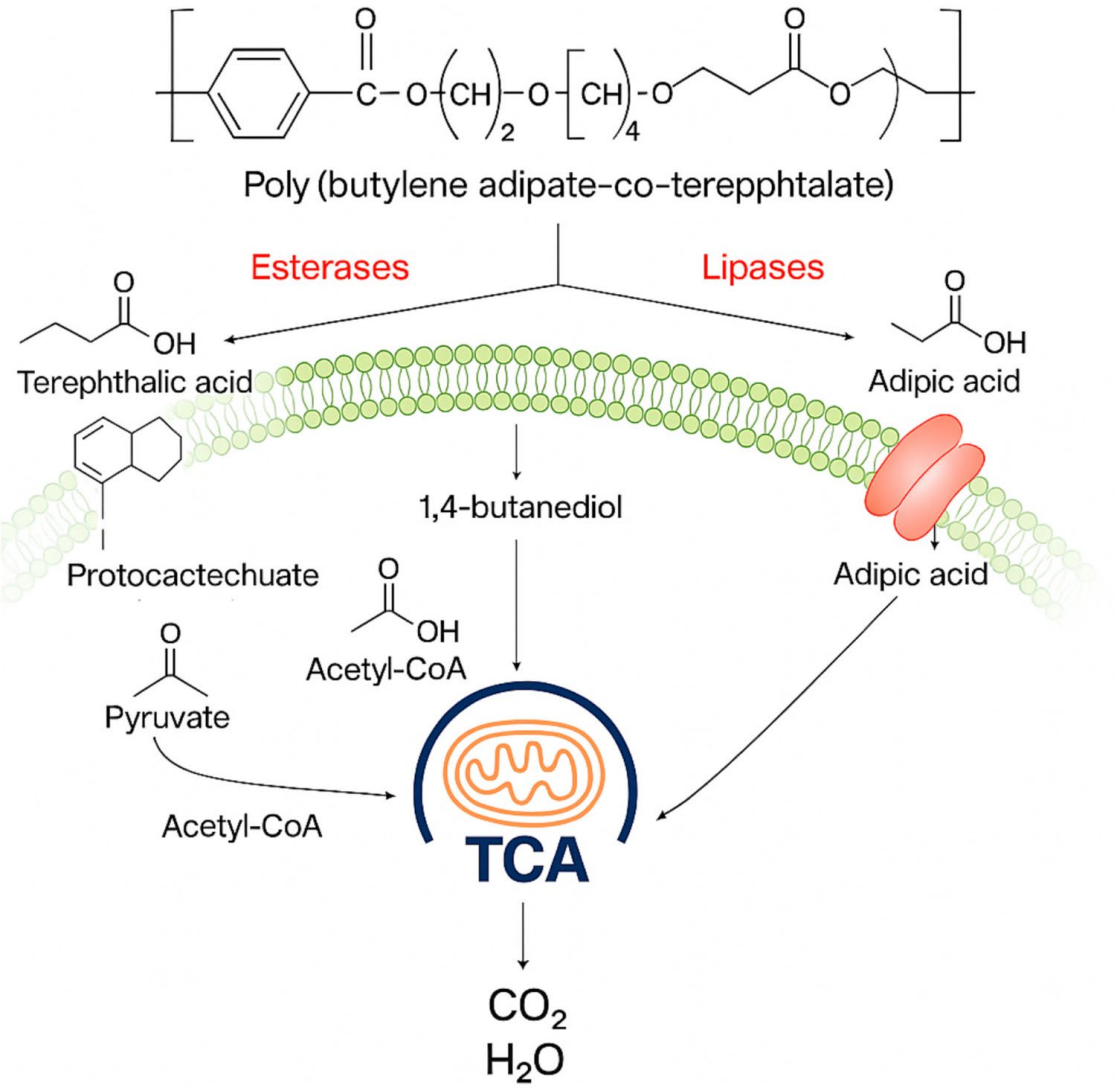
Common aerobic biodegradation pathway for PBAT, starting from the hydrolysis by esterases and lipases and ending on the tricarboxylic acid cycle (TCA) cycle (Bher et al. 2022)

The first step of PBAT biodegradation is the hydrolysis that can be catalysed, for example, by esterases, cutinases, lipases, and PBAT hydrolases (Fig. 1). The enzymatic hydrolysis of PBAT produces bis(4-hydroxybutyl) terephthalate and 1,4-butanediol that result from the hydrolysis of the aliphatic ester bonds (butylene adipate), as well as mono(4-hydroxybutyl) terephthalate and terephthalic acid resulting from the hydrolysis of the aromatic ester bonds (butylene terephthalate) (Müller et al. 2017). Several routes have been identified for the PBAT and PBAT monomer biodegradation. Terephthalic acid can be aerobically degraded using different degradation pathways. Normally, it is converted into protocatechuate via the metabolic funnel of aromatics that needs only a little dedicated enzymes as represented in Fig. 2 (Wang et al. 1995). Then, the protocatechuate is converted into pyruvate and further to acetyl coenzyme A (Daisuke et al. 2009). 1,4-Butanediol can also be oxidized to 4-hydroxybutyrate and then later to succinyl-CoA by oxidation, although it can be oxidated to succinate or beta-oxidated to glycolyl-CoA and acetyl-CoA (Li et al. 2020). Adipic acid can also be converted to adipyl-CoA by an adipate-CoA ligase and then further converted to acetyl-CoA (Strittmatter et al. 2022). The products resulting from the monomers can be introduced into the tricarboxylic acid cycle to create energy and release CO_2_ and water (Bher et al. 2022).

Normally, the biodegradation of the aliphatic ester bonds is faster, since they are soft and amorphous, contrary to the more rigid and crystalline aromatic ester bonds (Fu et al. 2020). Even so, not many microorganisms have been identified as able to degrade PBAT, but most of the strains belong to 3 phyla: *Actinobacteriota*, *Bacillota*, and *Pseudomonadota*. Table 2 shows the organism identified as capable of degrading PBAT. At thermophilic temperatures, *Thermomonospora fusca* and *Thermobifida alba* have been identified as initial degraders (Hu et al. 2010; Kleeberg et al. 1998; Thumarat et al. 2012, 2015). At mesophilic temperatures, *Bacillus subtilis*, *Bacillus pumilus*, *Leptothrix* sp. TB-71, *Peribacillus frigoritolerans* S2313, *Stenotrophomonas* sp. YCJ1 (isolated from farmland soil), *Bacillus* sp. JY35, *Bacillus* sp. JO01, *Rhodococcus* strain NKCM 2511, and *Rhodococcus fascians* NBRC 100625 were the aerobic bacteria that presented the capacity to degrade PBAT (Jia et al. 2021; Muroi et al. 2017; Nakajima-Kambe et al. 2009; Oh et al. 2025; Soulenthone et al. 2020; Trinh Tan et al. 2008; Wufuer et al. 2022; Yeon et al. 2022). A co-culture system with 3 bacteria isolated from soil was used to successfully degrade PBAT (Zhang and Sharaf 2024). A bacterium, *Roseateles depolymerans* TB-87, isolated from freshwater was reported as able to degrade aliphatic and aliphatic–aromatic copolyesters, including PBAT (Shah et al. 2013, 2014). More recently, the bacterium *Roseibium aggregatum* ZY-1 was also identified from water (Pan et al. 2024).

**Table 2:** Organisms and enzymes identified as capable of degrading PBAT

Enzyme	Microorganism	UniProt	EC number	References
Polyesterase	*Pseudomonas pseudoalcaligenes*	W6R2Y2	EC 3.1.1.74	(Wallace et al. 2017)
Hydrolase	*Rhodococcus fascians* NKCM251	A0A7I8E2Z4		(Soulenthone et al. 2021)
Cutinase	*Thermobifida cellulosilytica*	E9LVH8	EC 3.1.1.74	(Perz et al. 2016a)
		E9LVH9		
Cutinase	*Thermobifida alba*	D4Q9N1	EC 3.1.1.1.74	(Thumarat et al. 2015)
Cutinase	*Thermobifida alba*	F7IX06	EC 3.1.1.1.74	(Thumarat et al. 2012)
Hydrolase	*Pelosinus fermentans* DSM 17108	A0A0A0YMQ9		(Biundo et al. 2016)
Esterase	*Clostridium botulinum*	UPI0006BCBA3E		(Perz et al. 2016b)
		UPI0006BC7D7E		
Hydrolase	*Bacillus pumilus*	A0A1E1FNX8		(Muroi et al. 2017)
Polyesterase	*Saccharomonospora viridis*	W0TJ64		(Kawai et al. 2014)
Carboxylesterase	Uncultured *bacterium*	A0A1C9T884		(Müller et al. 2017)
Carboxylesterase	Uncultured* bacterium*	A0A1C9T772		
Carboxylesterase	Uncultured* bacterium*	A0A1C9T7D1		
Carboxylesterase	Uncultured* bacterium*	A0A1C9T784		
Carboxylesterase	Uncultured* bacterium*	A0A1C9T7D3		
Carboxylesterase	Uncultured* bacterium*	A0A1C9T7G6		
Cutinase	*Humicola insolens*	A0A075B5G4	EC 3.1.1.74	(Perz et al. 2016a)
Cutinase	*Fusarium solani*	P00590	Novozym® 51,032	(Zumstein et al. 2017)
Esterase	*Hungatella hathewayi* DSM 13479	UPI000731EA1F		(Perz et al. 2016c)
Hydrolase	*Thermobifida fusca* DSM 43793	Q6A0I3		(Kleeberg et al. 2005; Müller et al. 2005)
		Q6A0I4		
Cutinase	*Paraphoma* sp. B47-9	A0A060N399		(Suzuki et al. 2014)
Cutinase	*Saitozyma flava (Cryptococcus flavus)*	A0A0P0ZE81		(Watanabe et al. 2015)

Even in anaerobic environments, PBAT is biodegraded. The bacterium *Hungatella hathewayi* DSM 13479 (formerly known as *Clostridium hathewayi*) was found to be capable of degrading PBAT anaerobically (Perz et al. 2016a). Two PBAT hydrolases were also identified from the anaerobic mesophilic bacteria *Clostridium botulinum* ATCC 3502 and *Pelosinus fermentans* DSM 17108 (Biundo et al. 2016; Perz et al. 2016b).

Some strains of yeast and fungi have also been indicated as PBAT-degrading organisms, namely *Cryptococcus* sp. MTCC 5455 (phylum *Basidiomycota*), *Isaria fumosorosea* NKCM 1712 (phylum *Ascomycota*), *Purpureocillium lilacinum* BA1S, *Paraphoma*-related fungus cutinase-like enzyme, and *Cryptococcus flavus* cutinase-like enzyme (Aarthy et al. 2018; Kasuya et al. 2009; Sung et al. 2023; Suzuki et al. 2014; Watanabe et al. 2015). In previous works, it was also possible to isolate two fungi closely related to *Purpureocillium lilacinum* and *Aspergillus pseudodeflectus* capable of degrading PBAT (Fernandes et al. 2024). *Rhizopus oryzae* lipase and *Fusarium solani* cutinase are enzymes capable of degrading PBAT films with different terephthalate-to-adipate molar ratios (Zumstein et al. 2017). A fungal cutinase from *Humicola insolens* (HiC) and a bacterial cutinase from *Thermobifida cellulosilytica* (Thc_Cut1) were also able to hydrolyse the PBAT-tested esters (Perz et al. 2016c). About 6 enzymes of uncultured microorganisms in microbial communities associated with the *Sphagnum magellanicum* moss were able to degrade PBAT (Müller et al. 2017).

In aquatic environments that are not so well studied, a PBAT-hydrolysing enzyme has been described from *Pseudomonas pseudoalcaligenes*, a typical aquatic microorganism (Wallace et al. 2017). In a marine microbial enrichment culture, two enzymes were also identified in species from the genus *Marinobacter* sp*.* (Meyer-Cifuentes et al. 2020). As indicated before, several types of enzymes are capable of degrading PBAT, namely, cutinases, lipases, and PBAT hydrolases (Aarthy et al. 2018; Jia et al. 2021; Kasuya et al. 2009; Soulenthone et al. 2021; Zumstein et al. 2018). Nonetheless, the extracellular enzymes identified as capable of degrading PBAT are quite scarce in comparison to those for common aliphatic polyesters. It is important to mention that not all organisms or enzymes are capable of completely degrading all the components of PBAT, the aliphatic unit being much more susceptible to degradation than the aromatic unit. Some of these studies tested directly the efficiency of the enzymes, and very few studied the PBAT degradation in real or simulated environments, such as soil, compost, or water.

It seems clear that several types of microorganisms exist in different environments with the capability of degrading PBAT. However, most microorganisms cannot be cultured using standard lab techniques, so these methods limit the identification of potential PBAT degraders. Some innovations should be implemented to get better insights on the process, that include multi-analytical techniques such as LC–MS/MS to detect small molecular degradation products, or metagenomics and metatranscriptomics, to identify active microbial populations and gene expression during PBAT biodegradation. The knowledge about the already identified enzymes and microorganisms indicated here can be used in the development of engineered consortia to accelerate biodegradation, for example, for remediation solutions or creating new waste management solutions.

## PBAT biodegradation in the environment – the particular case of soil

PBAT is only considered compostable under typical compost conditions (Witt et al. 2001). It is considered resistant to biodegradation in natural environments (Han et al. 2021). However, some studies report PBAT biodegradation in soil. For example, pristine PBAT films could be partially biodegraded in soil but at a relatively slow rate (e.g., 21% in 180 days, 18% in 182 days, and 6% after 100 days) when compared with PHAs biodegradation (Palsikowski et al. 2018; Šerá et al. 2016; Souza et al. 2019). Even though the degradation of PBAT is considered slow, especially at mesophilic temperatures, it must be pointed out that Wang et al. (2015) noticed that antimicrobial PBAT composite films buried in soil were still biodegradable. In another study, it was reported that mechanical changes in PBAT in soil in only one month are much faster than in lab experiments, which take approximately 6 months, showing that other environmental factors influence positively PBAT degradation (Nikolić et al. 2017). Table 3 presents the studies about the PBAT biodegradation in soil.

**Table 3:** Biodegradation of PBAT in soil

Type of plastic	Type of material	Type of soil	Conditions test	Test method	Biodegradation (%)	Length of test (days)	Reference
PBAT	Film	Soil (unspecified)	25 °C	CO_2_ formation	8 to 13	42	(Zumstein et al. 2018)
PBAT	Film	Alluvial-type soil	35% soil moisture	Weight loss	20	120	(Wu 2012)
PBAT	Film	Soil (unspecified)	28 °C, 60% water holding capacity	CO_2_, ASTM D5988 (2012)	18 ± 4	182	(Souza et al. 2019)
PBAT	Film	Real soil burial conditions	5–20 °C	Weight loss	2.3	90	(Wang et al. 2015)
PBAT	Powder	Agricultural soil, sterilized, inoculated with fungal consortium (2 × 105 cells/ml)	30 °C, 80–100% of the moisture-holding capacity	CO_2_, ASTM D5988 (2003)	11	210	(Saadi et al. 2013)
PBAT	Film	Bulk soil	30 °C, 30% water	Weight loss	22	180	(Muroi et al. 2016)
PBAT	Film	Soil	25° C, 16.7 C/N ratio, pH 6.3, 60% retention capacity	CO_2_, ASTM D5988 (2012)	21	180	(Palsikowski et al. 2018)
PBAT	Film	Lou soil	30 °C, 60% water holding capacity	CO_2_	16	120	(Han et al. 2021)
		Fluvo-aquic soil			9		
		Black soil			0.3		
		Red soil			0.9		
PBAT	Film	Brown soil, black soil, and fluvisol	25° C, pH 6.45–6.85, 60% retention capacity	CO_2_	6	100	(Šerá et al. 2016)
PBAT	Film	Agriculture soil	12.5–25 °C, pH 7.9	Weight loss	89.8 ± 4.3	364	(Wang et al. 2025)
			16–21 °C, pH 7.7		92.3 ± 1.3		
			16–22.5 °C, pH 8.3		91.7 ± 2.0		
PBAT	Film	Agriculture soil	Buried at 10 cm	Weight loss, ASTM G160 (2012)	44.86	270	(Akhir et al. 2023)

The composition of the microbial communities in soil and even in composting processes is a crucial factor for achieving efficient biodegradation. Soil microbiomes contain a huge diversity of microorganisms capable of producing extracellular enzymes with hydrolytic activity. Thus, it is possible that suitable enzymes can be produced by several species for the same role in the biodegradation process (Šerá et al. 2020). Under composting conditions, the biodegradability extent/rate also varies much and has been attributed to the variability in microbial community composition (Kijchavengkul et al. 2010b). Different microorganisms have different metabolic potential and may or may not have enzymatic machinery to biodegrade PBAT, being more or less effective than others at biodegrading the polymer studied.

Several strains of genus *Bacillus* and class *Actinomycetes* present in soil and compost are capable of degrading PBAT (kanwal et al. 2022; Witt et al. 2001). Witt et al. (2001) indicated that *Thermomonospora fusca* DSM43793 can initially depolymerase PBAT but is not able to metabolize the monomers and oligomers formed. The *Bacillus* strains could completely degrade PBAT using lipases (kanwal et al. 2022). The relative abundance of *Bacillus* tends to increase during the biodegradation assays in lab experiments (kanwal et al. 2022; Witt et al. 2001). As indicated before, existing scientific knowledge indicates the strong possibility that PBAT degradation in soil involves the synergistic activity of different organisms. For instance, a co-culture of *Thermobispora bispora* (a thermophilic actinomycetes) and a *Bacilli* species (mostly of the genus *Geobacillus)* was found to biodegrade PBAT but could not carry out the biodegradation alone (Šerá et al. 2020). The isolation tested in this work may deprive the microorganisms of their necessary interactions and synergetic activity to accomplish the biodegradation (Šerá et al. 2020). The diversity and composition of the soil community may thus influence biodegradation because other microorganisms might not have enzymes capable of degrading directly PBAT but may contain enzymes involved in the downstream pathway of degradation and are able to catabolize intermediates of PBAT degradation, thus acting synergistically. In lou, black, and red soil tested in the laboratory, differences in the biodegradation of films were obtained (0.3 to 16%), due to microbial community differences (Han et al. 2021). Šerá et al. (2020) could not find mesophilic PBAT degraders in 41 temperate zone soils and found a low number of thermophilic degraders in only nine soils, although some more were found after an enrichment process. This indicates that the complete biodegradation of blends or composites with PBAT in their composition may be slow due to the shortage of microorganisms able to degrade it. In short, it is crucial to analyse the biodegradation of these blends and composites, because some formulations can be degraded at a faster rate than pristine PBAT films (Šerá et al. 2016). Finally, in real environments, some microorganisms may act as initial PBAT degraders, and others may act as commensal organisms, using monomers and/or other degradation products, having also an essential activity.

To better understand the process in real scenarios, dynamic soil reactors to simulate real conditions with controlled aeration, humidity, and temperature may be used; soil mesocosms to study long-term degradation under semi-natural conditions could also be valuable. Connecting biodegradation data with geochemical and climate models may be very helpful for predicting PBAT persistence and behaviour in real environments (marine or terrestrial).

## PBAT properties affecting biodegradation

The aliphatic chains in PBAT exhibit high biodegradation rates when compared with the PBAT aromatic rings (Marten et al. 2005). The flexibility and mobility of the PBAT aliphatic chains facilitate the binding of the enzymes (Marten et al. 2005). The polymer chemical structure influences the spatial arrangement, resulting in the formation of different regions (amorphous and crystalline) (Muroi et al. 2017). The PBAT crystallinity plays a major role, since normally crystalline regions are more rigid and stiff and consequently are less prone to degradation, while the amorphous regions are more susceptible to hydrolysis (Kijchavengkul et al. 2010b). The mass loss of PBAT with 43% of BT unit or 61% after 56 days in soil was 4.94% and 1%, respectively. According to the authors, this may be caused by an increase in the average sequence length of the BT unit and in the improvement of the crystallinity and melting point, which makes difficult the diffusion of water molecules and hydrolysis reactions (Zhang et al. 2023). The molecular weight also influences biodegradation, since the higher the molecular weight, the harder it is for microorganisms to bio-assimilate PBAT chain sections, which decreases the biodegradation rate (Kijchavengkul et al. 2010b). Souza et al. (2019) reported a mineralization of 18 ± 4, 14 ± 5, and 16 ± 5% for PBAT and PBAT with Carbon Black/Hindered Amine Light Stabilizer (CB/HALS) and Carbon Black/Vitamin E (CB/VE), respectively. The authors linked the results with the lower molecular weight (CB/HALS > CB/VE > PBAT) which benefits the flexibility of the materials and the diffusion of water. The size and shape of the polymers influence the surface area of the material, which is important since the biodegradation starts on the surface (Šerá et al. 2016). For example, PBAT with 25% starch-containing biodegradable plasticizers presented an increased biodegradation, from 6% for neat PBAT to 53% in 100 days, according to the authors due to an increase in the active surface area after the biodegradation of the filler (Šerá et al. 2016). PBAT was among the most degradable polymers in uncontrolled composting conditions, and all of them presented very rough surfaces as compared to the other polymers (Mercier et al. 2017). The roughness has an impact on biodegradation since irregularities provide places for microbial attachment and therefore favour microorganisms-polymer contacts that may increase polymer degradation (Mercier et al. 2017).

To conclude, the intrinsic PBAT properties that influence its biodegradation more are the crystallinity; it is inherently hydrophobic, since more hydrophilic films absorb water more easily, accelerating hydrolysis and microbial degradation. This can be changed with the use of additives or blending with hydrophilic polymers (Šerá et al. 2016). The aromatic ring, existing in the BT unit, also influences the biodegradation, since more aromatic content makes biodegradation more difficult (Marten et al. 2005).

## Biodegradation of PBAT blends or composites

The use of PBAT with other polymers or with naturally decomposable materials such as natural fibres can be used as a strategy to reduce production costs or change the properties according to the goals established. Dammak et al. (2020) created a blend of PBAT with plasticized thermoplastic starch (TPS) and included suitable compatibilizers (e.g., maleic anhydride) for packaging applications. PBAT/TPS composites have lower prices but frequently present poorer mechanical properties due to the incompatibility between PBAT and TPS (Liu et al. 2020). Wei et al. (2015) used a compatibilizer (Joncryl-ADR-4368) or a synthesized styrene-maleic-anhydride-glycidyl methacrylate (SMG) reactive compatibilizer with the PBAT/TPS blends, and these blends presented enhanced mechanical performances appropriate for several applications, such as packaging and agriculture mulching films. Another frequent polymer blended with PBAT is PLA; nevertheless, PLA/PBAT blends present multiphase behaviour because of the non-compatible attributes of each element, which produces weak mechanical properties (Sarath Kumara et al. 2008); Jiang et al. 2006) reported alterations from brittle fracture to ductile fracture in tensile testing with increased PBAT fraction and low interfacial adhesion. PBAT/PLA films developed for agricultural purposes (mulch films), with 10–20% in weight of calcium carbonate, presented enhanced compatibility with good maximum strain, tensile strength, and Young’s modulus (Rocha et al. 2018). The addition of Joncryl and 1,6-hexanediol diglycidyl ether to the blends has been reported to improve up to almost 500% of the strain at break (Dong et al. 2013). Other well-known PBAT blends are created with lignin. Xiong et al. (2020) produced PBAT/lignin blends via melt extrusion with up to 60% lignin content. Although the authors reported a cost reduction of about 36%, the tensile (23.70 to 14.41 MPa) and elongation (816.49% to 378.94%) properties were also reduced. The addition of methylated lignin (60%) to PBAT increased these properties in relation to the PBAT/lignin films, but they were still inferior to the pristine PBAT films (Xiong et al. 2020). According to most of these studies, the use of PBAT with other polymers works better from the point of view of mechanical characteristics, when additives that increase compatibility between the polymers are used.

The development of new composites and blends also needs validation of the biodegradation behaviour, since if the properties of the initial materials can be changed, the biodegradation potential can also be affected. PBAT/lignin-blended films presented a decrease in mechanical properties after soil biodegradation when compared with neat PBAT (Liu et al. 2021). Films of PCL blended with PBAT presented a superior biodegradation (evaluated by mass loss) after 119 days in soil (37%) than solely PBAT (2.3%); however, the biodegradation was inferior to the PCL films (57%) (Sousa et al. 2022). This test was conducted between 30 and 35 °C. The PCL incorporation did not negatively affect all the properties including the tensile properties, indicating to the authors that the blends’ higher permeability can make the films useful in several applications. PBAT films with calcium carbonate (CaCO_3_) nanoparticles as nanofiller were incubated at 30 °C in soil. The CaCO_3_ nanoparticles decreased surface wettability and hindered the disintegration. The SEM analysis after the soil test revealed selective zones of disintegration (Rapisarda et al. 2022). PLA/PBAT blends are a little more studied, and sometimes fillers or compatibilizers are used to enhance the properties. Graphene-modified composite of PLA/PBAT presented different behaviours depending on the graphene content. The FTIR analysis indicated the priority degradation of PLA. With 0.2% of graphene (in weight), the degradation was inhibited, but with 1.0%, the degradation increased (Liu et al. 2022). The incorporation of montmorillonite clay in PLA/PBAT films resulted in a slower biodegradation in soil (28 °C); probably montmorillonite enhanced the hydrolysis by enabling water penetration but delayed the diffusion of oligomers to be used by microorganisms (Freitas et al. 2017). PLA/PBAT blends compatibilized with a chain extender (Joncryl ADR-4368) exhibited an intermediate behaviour compared to neat polymers; the authors indicated an increased crystallinity in the blend as one factor that influenced the biodegradation of the blends (Palsikowski et al. 2018).

Ultimately, PBAT composites and blends may present superior or inferior biodegradation compared to the neat polymer, depending on the properties modified. In the case of increased crystallinity or reduced water absorption, for example, the biodegradation tends to be slower. If the permeability is higher or the hydrophilicity increased, the biodegradation is normally enhanced. However, if the phase dispersion is poor, uneven biodegradation, by zones for example, may be verified. Understanding compatibility, microstructure, and environmental exposure is key to designing PBAT-based materials with better degradation profiles but also mechanical properties.

## Conclusion and future perspectives

PBAT shows the potential to be biodegraded in a relatively short period of time in environmentally relevant conditions, especially when compared with most of the polymers extensively used worldwide. PBAT is a primarily petroleum-based polymer, so future work should focus on the development of bio-based PBAT from renewable feedstocks and explore green synthesis pathways with lower carbon footprints since some but not sufficient work was done in that direction. In relation to disposal, its biodegradation in soil is slower than desired since there are not so many microorganisms known as PBAT degraders; therefore, its plastic containing waste should be separated and treated by efficient treatment strategies such as industrial composting. In future work, the improvement of PBAT formulations to enhance degradation across multiple environments, the development of enzyme-sensitive linkages or bio-triggered additives, and the creation of blends or coatings for targeted degradation behaviour are strategies that may be explored. Furthermore, the development of chemical recycling or enzymatic depolymerization methods or the improvement of the compatibility with other waste streams (e.g., compostables and mixed plastics) are also possible pathways. The knowledge about the microorganisms and/or their enzymes described here is crucial for developing these efficient biotechnological solutions for the treatment of waste containing PBAT at mesophilic temperatures, thus contributing to environmental cleaning. The disposal instructions for PBAT and other solutions should be clear to avoid creating yet another problem when trying to solve the problem of conventional plastics. The increase in consumer education is necessary to not create misunderstandings about what biodegradable and bioplastics truly means.

## Data Availability

No datasets were generated or analysed during the current study.
